# Fluorescent labeling strategies for molecular bioimaging

**DOI:** 10.1016/j.bpr.2025.100200

**Published:** 2025-02-12

**Authors:** Marcel Streit, Made Budiarta, Marvin Jungblut, Gerti Beliu

**Affiliations:** 1 Rudolf Virchow Center, Research Center for Integrative and Translational Bioimaging, University of Würzburg, 97080 Würzburg, Germany

## Abstract

Super-resolution microscopy (SRM) has transformed biological imaging by circumventing the diffraction limit of light and enabling the visualization of cellular structures and processes at the molecular level. Central to the capabilities of SRM is fluorescent labeling, which ensures the precise attachment of fluorophores to biomolecules and has direct impact on the accuracy and resolution of imaging. Continuous innovation and optimization in fluorescent labeling are essential for the successful application of SRM in cutting-edge biological research. In this review, we discuss recent advances in fluorescent labeling strategies for molecular bioimaging, with a special focus on protein labeling. We compare different approaches, highlight technological breakthroughs, and address challenges such as linkage error and labeling density. By evaluating both established and emerging methods, we aim to guide researchers through all aspects that should be considered before opting for any labeling technique.

## Why it matters

Understanding the details of fluorescent labeling is crucial for researchers employing super-resolution microscopy to study biological processes at the molecular level. As imaging techniques achieve resolutions approaching the molecular scale, the choice of labeling strategies directly impacts the accuracy and reliability of the observations. By staying informed about the latest developments in fluorescent labeling, scientists can enhance the accuracy of their experiments, leading to deeper insights into cellular functions and interactions that are fundamental to biotechnology and biomedicine.

## Introduction

Super-resolution microscopy (SRM) has revolutionized biological imaging by revealing cellular structures beyond the diffraction limit of conventional light microscopy. By circumventing this limit—which restricts resolution to about half the wavelength of the emitted light—SRM enables the visualization of biological processes at the molecular level, significantly deepening our understanding of cellular structures, interactions, and dynamics. As different SRM techniques have been developed over the past decades, each of these techniques has its own advantages and limitations, and the choice depends on the specific requirements of the study, such as resolution, imaging speed, sample preparation, and phototoxicity. Recent literature provides comprehensive reviews and guidelines to help researchers select the most appropriate SRM technique for their purposes (1,2,3).

At the heart of fluorescence microscopy is fluorescent labeling—the process of attaching fluorescent molecules to target biomolecules to enable their detection and visualization (4). A variety of labeling techniques now allow precise tagging and measurement of molecular interactions, dynamics, and spatial distributions at the nanoscale. The choice of fluorophores and the optimization of labeling density are crucial, as they influence resolution, specificity, and overall experimental success. Achieving optimal labeling density is critical for balancing resolution and specificity, as both excessive and insufficient labeling can compromise image quality (5,6). The integration of chemical synthesis and genetic engineering has led to novel labeling strategies, enhancing the versatility of fluorescent labeling. This interdisciplinary approach complements traditional methods such as classical immunostaining and has led to innovative techniques that exploit molecular specificity and affinity (7). In addition, the selection of appropriate fluorophores is essential, as each has unique biochemical and photophysical properties, providing flexibility to adapt to various experimental conditions (8). Novel bioconjugation technologies such as bioorthogonal click chemistry allow molecular tagging by enabling chemical labeling and visualization of biomolecules with minimal perturbation, high reaction speed, and reduction of steric demand (9,10).

In this review, we discuss recent fluorescent labeling strategies in the context of SRM, comparing different approaches and highlighting technological breakthroughs. Our aim is to explore the complexities of fluorescent labeling to highlight the technological advances and strategic considerations that are shaping this field, and to point toward future trends and applications. The successful application of SRM in cutting-edge biological research will depend on careful consideration of fluorescent labeling and rely on its continuous innovation and optimization.

## Overview of SRM techniques

To appreciate the advances in fluorescence labeling strategies, it is important to understand the different SRM techniques and their evolution. While each uses different strategies to achieve higher resolution, they all share the common goal of revealing details at the nanoscale (Table 1). The impact of fluorescent labeling becomes increasingly important as the achievable spatial resolution approaches the molecular scale. Especially techniques that achieve resolutions of 20 nm or better are particularly affected by the choice and optimization of labeling methods, as spatial resolution approaches the size of the target molecules themselves.

**Table 1:** Labeling strategies for SRM

Labeling strategy and label size	SRM techniques	Resolution impact	Target accessibility	Advantage	Limitation	Reference
Antibodies; nanobodies and Fab fragments 150 kDa; ∼15 kDa	STED SIM SMLM ExM	10–30 nm	◒	• high specificity • widely available • robust and established method	• large size increases linkage error • may block epitopes or inhibit function • often not suitable for live-cell imaging (especially difficult for intracellular targets) • reduced labeling density	Thevathasan et al. (11); Willig et al. (12); Sieber et al. (13); Kittel et al. (14); Willig et al. (15) Gustafsson et al. (16); Nixon-Abell et al. (17) Dani et al. (18); van de Linde et al. (19); Löschberger et al. (20); Winterflood et al. (21); Ehmann et al. (22) Chen et al. (23); Thevathasan et al. (11); Dani et al. (24)
Other structure-affinity binders (small molecules, affimers, aptamers, etc.) 10–18 kDa	STED SIM SMLM ExM MINFLUX	5–20 nm	●	• small size reduces linkage error • better tissue penetration • high stability • efficient labeling of abundant targets	• limited target availability • modulation of biological function • accumulation inside cells • limited commercial availability	Opazo et al. (25); Lukinavičius et al. (26); Mitronova et al. (27); Wong-Dilworth et al. (28); Bucevičius et al. (29) Takakura et al. (30); Gregor et al. (31); Huang et al. (32) Heilemann et al. (33); Tiede et al. (34); Wombacher et al. (35); Adhikari et al. (36); Shim et al. (37) Wen et al. (38) Gerasimaitė et al. (39)
Fluorescent proteins 25–35 kDa	STED SIM SMLM ExM	10–30 nm	◒	• genetically encoded; no staining required • ideal for live-cell and whole-organism imaging • versatile applications	• lower brightness and photostability • potential oligomerization affect function • moderate size increases linkage error	Sieber et al. (13); Hein et al. (40) Kner et al. (41); Li et al. (42); Guo et al. (43) Betzig et al. (44); Thevathasan et al. (11); Wang et al. (45) Chen et al. (23); Tillberg et al. (46)
Self-labeling enzymes 20–30 kDa	STED SIM SMLM ExM	10–20 nm	◒	• covalent or noncovalent, specific labeling • suitable for live-cell imaging • wide range of fluorophores • rapid labeling kinetics	• requires optimization to reduce background • ligand penetration and permeability • moderate size increases linkage error	Kompa et al. (47); Wong-Dilworth et al. (28); Lukinavičius et al. (48) Guo et al. (43); Holtmannspötter et al. (49) Thevathasan et al. (11); Kompa et al. (47); Lukinavičius et al. (48); Grimm (50); Barlag et al. (51) Shi et al. (52)
Affinity tags (protein/peptide tags) mostly >2 kDa	STED SIM SMLM ExM	2–15 nm	●	• precise motif-specific labeling • enables tracking and trafficking studies • reversible binding possible	• inefficient substrate delivery • potential for nonspecific interactions • possible cytotoxicity	Benaissa et al. (53) Tyler et al. (54) Chamma et al. (55); Lelek et al. (56); Mazloom-Farsibaf et al. (57) Trinks et al. (58)
Genetic code expansion and bioorthogonal chemistry ∼1 kDa	STED SIM SMLM ExM MINFLUX	1–5 nm	●	• minimal linkage error due to small label size • site-specific labeling • minimal perturbation of protein function • compatible with various fluorophores	• requires genetic engineering expertise • variable efficiency and expression levels	Arsić et al. (59); Saal et al. (60) ; Uttamapinant et al. (61); Jungblut et al. (62) Beliu et al. (63); Nikić et al. (64); Jungblut et al. (62); Neubert et al. (65) Sun et al. (66); White-Mathieu et al. (67) Mihaila et al. (68)

Summary of labeling strategies used in SRM, label size, resolution impact, target accessibility, advantages, limitations, and relevant references. Target accessibility properties are indicated by symbols (●, very high; ◒, high; ○, moderate).

SRM methods can be broadly grouped into deterministic or stochastic techniques, although overlaps exist, and strict categorization may not capture the nuances of each technique. In the following, we briefly mention the characteristics of the most used SRM techniques for biological imaging:

Structured illumination microscopy (SIM) achieves super-resolution by illuminating the sample with modulated excitation light, producing interference patterns at the sample plane. This interference pattern convolves the fluorescent signal of the stained structure, shifting high spatial frequency information into lower, observable frequencies (a process analogous to Moiré fringes) (69). By capturing images with different pattern orientations and phase shifts, a super-resolved image can be reconstructed computationally. SIM thus improves the resolution by a factor of two over conventional microscopy, reaching 60–200 nm resolution (16). SIM is well suited for live-cell imaging, especially dynamic processes like cytoskeletal movements, intracellular transport, or membrane trafficking due to its relatively low phototoxicity and rapid acquisition times, but light scattering in thick samples may cause interferences with the structured illumination pattern (70).

Stimulated emission depletion (STED) microscopy enhances resolution by selectively depleting fluorescence in specific regions using a doughnut-shaped depletion laser aligned with the excitation beam (71). This process effectively reduces the size of the point spread function, allowing resolutions down to 60–100 nm (72). STED is, in principle, suitable for live- and fixed-cell imaging due to its ability to achieve high spatial resolution in real time, especially in neuroscience and cell biology, although the requirement for high-intensity lasers can lead to photobleaching and phototoxicity (73,74).

Single-molecule localization microscopy (SMLM) techniques, including PALM, *d*STORM, and DNA-PAINT, achieve high spatial resolutions—nowadays reaching 20 nm or better—by precisely localizing individual fluorescent molecules and reconstructing the overall structure from these positions (75). In PALM, photoactivatable fluorescent proteins (FPs) are typically used, while *d*STORM utilizes organic fluorescent dyes that can reversibly switch between fluorescent and dark states under specific buffer conditions (44,33). DNA-PAINT leverages the transient binding of fluorescently labeled DNA strands to complementary target strands, allowing for high localization precision down to 1–5 nm resolution (76) and multiplexing capabilities (77). This is advantageous for densely packed structures like nuclear pores or cytoskeletal targets and circumvents problems like photobleaching or the need of optimal photoswitching. MINFLUX nanoscopy combines elements of SMLM and STED to achieve localization precisions in the range of 1–5 nm (78). By utilizing minimal photon flux and a doughnut-shaped excitation beam with a central intensity minimum, MINFLUX can localize fluorophores with high accuracy, approaching the size of the fluorophore itself (79). While MINFLUX faces the similar challenges as other SMLM techniques (e.g., requiring photostable dyes and appropriate labeling densities), its high localization precision enables accurate molecular tracking (80). However, achieving such high resolution intensifies the challenges associated with fluorescent labeling, as any labeling artifacts or photophysical effects can significantly impact localization accuracy and probability (81). As novel DNA-barcoding technologies can reach sub-nm localization precision, the impact of labeling becomes increasingly critical (76).

Contrary to the abovementioned SRM techniques, expansion microscopy (ExM) offers a complementary approach by physically expanding the biological specimen embedded in a swellable hydrogel, effectively increasing the distance between molecules (23). When optimized, ExM can achieve molecular resolution and has become a valuable tool for molecular bioimaging. ExM is compatible with conventional microscopes, making it accessible to a wider range of laboratories (82). However, effective cross-linking and labeling are crucial, as fluorophores or labels must be anchored to the polymer network to ensure accurate representation of molecular structures after expansion (46). Inefficiencies can lead to distortions or loss of information, highlighting the importance of optimized labeling strategies even in ExM (83).

## Today’s demands to SRM

SRM techniques share common requirements with all fluorescence-based microscopy techniques but face unique challenges due to their advanced capabilities. To meet diverse and growing applications, it is essential to address the specific needs driving advances in SRM. These advancements depend on several critical areas, including.

(1) Technological progress: achieving higher resolution necessitates continuous innovation in both hardware (such as lasers, detectors, optical components, and computational power) and software for image processing and analysis.
(2) Sample preparation: maintaining biological integrity is fundamental. Methods must preserve cellular and molecular structure and function while minimizing potential artifacts, ensuring the authenticity of observed structures and dynamics.
(3) Innovations in fluorescent labeling: developing advanced labeling techniques enhances the precision and efficiency of tagging specific molecules, improving imaging resolution while minimizing disruption to biological systems.
(4) Fluorophore selection: a diverse range of fluorophores is necessary to accommodate various experimental conditions and applications, emphasizing properties such as spectral characteristics, photostability, and photomodulation.
(5) Operational flexibility: SRM increasingly requires the ability to perform multicolor imaging to visualize multiple targets simultaneously.
(6) Accessibility and ease of use: SRM systems should feature user-friendly interfaces accessible to nonexperts and be cost-effective to ensure broader availability across laboratories.

Sample preparation and labeling strategy are closely linked and often intertwined in terms of timing and technology (84). In the past, whenever novel sample preparation protocols, labeling technologies, or imaging methodologies were introduced, reference structures with known dimensions, well below the diffraction limit of standard fluorescence microscopes, were used to validate resolution capacities. These included the nuclear pore complex with its wheel-shaped, eightfold symmetrical structure (∼125 nm diameter); microtubules (∼25 nm outer diameter); the synaptic cleft in chemical synapses (15–25 nm separation between membranes); and DNA origami nanostructures, which, due to their precisely controllable dimensions, served as super-resolution imaging rulers (11,18,85,86,87). With the trend toward molecular imaging, artificial DNA structures such as DNA nanorulers and DNA origami have gained popularity due to their ease of synthesis and ability to be labeled with dyes at precise locations. While they have been instrumental in demonstrating the capabilities of SRM, particularly in achieving sub-20 nm spatial resolution (76,79,88), their usefulness for cellular imaging remains controversial (81). Ideally, biologically relevant reference structures such as multiprotein complexes should be used to evaluate new SRM methods, as these represent the target structures in biomedical applications. However, challenges arise due to the size, steric hindrance, and significant binding errors associated with conventional fluorescent probes. Recent developments in protein-based reference systems, which can be site specifically and quantitatively labeled with small fluorophores, are expanding the toolbox for high-precision imaging and represent a valuable alternative for optical reference systems (81,24).

## Localization precision and structural resolution

**Figure 1:**
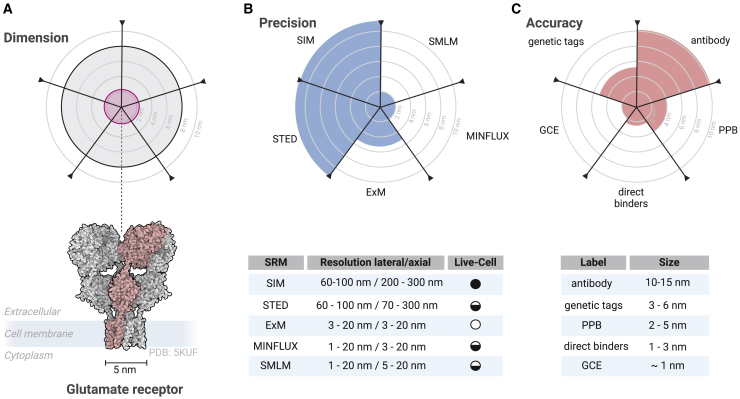
The critical role of selecting appropriate SRM techniques and labeling strategies for successful target visualization. (*A*) Structural representation of the glutamate receptor. The oligomeric form is shown in gray, and a single protomer is highlighted in red. A simplified top-view spider-chart illustrates the spatial dimensions of the receptor. The distance between two gray lines corresponds to 2 nm. (*B*) Imaging precision of various SRM techniques. The achievable localization precision (*blue*) for different SRM methods, indicating the potential resolution relative to the receptor structure. (*C*) Imaging accuracy influenced by labeling tags. The effective imaging accuracy (*red*) is depicted based on the sizes of different labeling tags.

In SMLM, the ability to resolve biological structures is fundamentally linked to localization precision and accuracy. Achieving high structural resolution requires not only precise localization, but also optimized labeling strategies that enhance this precision through tailored and targeted fluorescence signals. The choice of labeling approach can therefore directly influence the ability to accurately visualize biological architectures at the molecular level (Fig. 1).

A critical factor affecting localization accuracy is the linkage error. In general, as soon as the spatial resolution is approaching the size of the target biomolecules, the size of fluorescent tags and the linkage error will restrict achievable structural resolution (82,89,90). Several factors contribute to the linkage error: first, the size of the label. Larger labels, such as fluorophore-conjugated antibodies (∼10–15 nm), introduce a substantial distance between the target molecule and the observed fluorescence signal, potentially obscuring structural details, although linkage errors from primary and secondary antibodies do not add up (90). Secondly, the accessibility of the binding site plays a crucial role; attaching fluorophores to sites distant from the functional or structural region of interest can misrepresent the position and orientation of the molecule, making it difficult to accurately reconstruct the architecture of the sample. Label orientation, the exact number of fluorophores, and labeling efficiency are additional critical parameters for optimal fluorescent labeling. While the localization accuracy of fluorophore-labeled targets is not necessarily dictated by the number of fluorophores bound, high labeling density is important for resolving complex biological structures. In relation to sampling theory, the density of fluorescent probes must be sufficient to ensure that the average distance between neighboring fluorophores is at least half of the desired spatial resolution (91).

Quantitative labeling—the ratio of labeled target molecules to all biomolecules of the same type—also has a direct impact on imaging quality. Achieving high labeling efficiency is challenging due to several biological limitations. Low abundance of target molecules can make it difficult to achieve sufficient labeling density without causing nonspecific binding. Labels can interfere with the natural behavior or localization of the target molecule, introducing artifacts or compromising functionality. In addition, inefficient or disruptive delivery mechanisms, such as transfection or permeabilization, can result in incomplete or uneven labeling. It is essential that fluorescent labeling is uniform and representative of the entire population, as inhomogeneous labeling leads to biased or incomplete structural views, which are particularly problematic in densely packed cellular environments where precise spatial arrangements and molecular interactions are important.

For quantitative analysis, stoichiometric labeling is crucial. Especially site-specific labeling using genetic code expansion (GCE) and click labeling or self-labeling tags can ensure a one-to-one ratio of fluorophore to target. For SRM techniques that achieve resolutions of 20 nm or better, the physical size of the label and the linkage error can introduce significant bias. Nonoptimized labels can lead to mislocalization of target molecules, ultimately leading to misinterpretation of molecular arrangements and interactions. Optimization of labeling strategies is essential to achieve accurate and reliable molecular bioimaging. While all SRM techniques benefit from effective labeling, those that achieve resolutions of 20 nm or less are particularly sensitive to labeling artifacts. Ultimately, optimizing labeling density and efficiency not only improves localization accuracy, but also advances nanoscale resolution and ensures reliable molecular bioimaging.

## Labeling strategies and fluorescent probes

The quest to elucidate cellular and molecular structures led to the development of a diverse range of fluorescent labeling strategies. These strategies can be broadly divided into two main types: those exploiting specific affinity between a label and its target (structure-affinity labels) and those employing genetic modifications to directly label proteins or other biomolecules of interest (genetic modification labels). Each approach offers distinct advantages and presents unique challenges, necessitating careful consideration of application-specific factors.

### Structure-affinity labeling

Structure-affinity labels have been central to fluorescence microscopy, providing robust means of localizing and visualizing molecular targets in complex biological specimens (7). They bind specifically to target molecules, enabling selective labeling without prior genetic modification, which was a key advantage for studies requiring unmodified visualization of cellular components.

### Antibodies and nanobodies

Among the numerous structure-affinity labels, antibodies, especially immunoglobulin G, serve as cornerstones in fluorescence microscopy (92). These Y-shaped proteins can recognize and bind a variety of targets with remarkable specificity (93). Their versatility extends beyond imaging, as they are also integral to detection assays and purification methods. However, the relatively large size of antibodies (∼150 kDa) can limit access to target sites, reduce labeling density, and increase linkage errors, potentially obscuring true structural details. In addition, their interaction with active epitopes can impair the functional integrity of the target molecule (84). Therefore, careful optimization of immunostaining protocols is essential to minimize artifacts, especially in procedures involving cell fixation and permeabilization (94). Despite these limitations, antibodies remain indispensable in SRM. Techniques like ExM have taken advantage of the robustness of antibodies, enabling visualization of biological structures at nanoscale resolution using conventional fluorescence microscopes (23). Whenever antibodies are not the primary choice, smaller protein binders such as nanobodies, monobodies, peptide, or protein binder (PPB) offer a compelling alternative (95,96,97,98). Significantly smaller (<15 kDa) than conventional antibodies, peptide and other protein binders provide better penetration into inaccessible epitopes, reducing linkage error and improving labeling density. Their enhanced chemical and thermal stability, coupled with improved tissue penetration and ease of modification, make them suitable for high-resolution imaging applications. PPBs have been successfully used to target FPs and tags, facilitating high-resolution imaging with reduced linkage error (99). Their application spans a wide range of cellular structures and has spurred the development of PPB-based reporters for specialized targets (100). One example is affimers. Derived from synthetic protein scaffolds, affimers are recombinant affinity reagents that mimic antibodies in their binding properties but are significantly smaller (∼12–14 kDa). They offer high specificity and affinity, providing a robust and thermally stable platform for various labeling applications (34,101). Although their commercial availability is currently limited, affimers have been successfully used to label cellular components such as the actin cytoskeleton and tubulin (101,102). Another class of protein-based binders are designed ankyrin repeat proteins. These target-specific binders are of similar size as nanobodies (14–18 kDa) but do not contain sulfide bonds and bind with high affinity (103). Generally, their epitope-specific labeling capabilities make PPBs versatile tools in super-resolution imaging, as demonstrated by their application in various SRM techniques (102,104).

### Aptamers

Aptamers are single-stranded DNA or RNA molecules that fold into specific three-dimensional structures, enabling them to bind proteins, peptides, and small molecules with high affinity and specificity (25,105). RNA aptamers have been instrumental in developing RNA-fluorophore complexes for fluorescent biosensors, while DNA aptamers have found applications in techniques like DNA-PAINT as specific and tunable markers (77). As an example, the subclass of slow off-rate modified aptamers exemplifies high-affinity binding, making them well suited for SRM (106). A novel combination of aptamers with a rhodamine dye, called RhoBAST, enables SMLM with fast kinetics and reduced photobleaching (107).

### Small organic probes

Small organic probes encompass a variety of molecules designed for high-affinity binding to specific targets (4). Their small size allows them to penetrate cells easily, making them suitable for intracellular studies (108). These labels are often preconjugated to fluorescent dyes and can bind directly to endogenous targets, offering options for staining cellular structures without the need for genetic modification or complex labeling protocols.

Recent advances have leveraged small-molecule fluorescent probes for direct targeting of cellular components such as DNA, cytoskeleton, lipids, and organelles. Developments like exchangeable dyes for STED imaging of bacterial cells and membrane dynamics underscore ongoing innovation in this field of research (30). Small organic probes also serve as sensors for calcium, pH, and voltage, highlighting their utility in functional fluorescence microscopy (8). Novel techniques such as traceless affinity markers and affinity-driven catalytic labels expand the applications of fluorescence microscopy, illustrating the dynamic evolution of structure-affinity labels in super-resolution imaging (109,110,111).

## Genetic modification labeling

When affinity-based labels are not available or applicable, genetic engineering provides a versatile alternative (112). This approach involves incorporating or attaching fluorescent molecules directly into proteins of interest (POIs). Strategies range from integrating entire FPs or peptides to shorter modifications like peptide motifs and even single amino acids. Site-specific attachment of fluorophores enhances accuracy and selectivity, enabling detailed visualization and tracking of molecular interactions in living cells. Often, that integration of nonendogenous proteins into cells is done via overexpression. This can lead to nonphysiological or toxic concentrations and heterogenic expression levels between cells. An unusual high concentration of target protein also interferes with the endogenous distribution of protein inside the cell and may hamper its function via accumulation. Besides that, cells with overexpressed proteins may have decreased viability due to lack of resources for translation of endogenous proteins. Potential strategies to circumvent this problem include the generation of stable cell lines, inducible expression systems, or self-regulating promotor systems.

### FPs

Integrating FPs into the protein sequence represents a direct and widely used genetic engineering strategy for SRM (113). The pioneering use of green FP transformed cellular imaging by allowing observation of protein targets and their interactions within their native cellular environment. FPs facilitate the production of fusion proteins directly in cells, eliminating the need for external staining procedures and simplifying live-cell imaging. Despite their utility, FPs come with limitations. Their relatively large size (∼25–35 kDa) may affect the function or localization of the POI. In addition, their photophysical properties may not be optimal for all super-resolution techniques, particularly those requiring photoswitching or high photostability. Advances in FP engineering have led to a range of derivatives suitable for multicolor imaging, such as red fluorescent proteins (e.g., mCherry, mScarlet) and improved green variants (e.g., mEmerald, mNeonGreen, mBaojin) (114,115,116). Photoswitchable and reversibly photoswitchable FPs have been developed to mitigate photobleaching and broaden the scope of super-resolution and correlative SRM-electron microscopy applications (117,118,119,120).

### Protein tags and self-labeling enzymes

Protein tags and self-labeling enzymes are essential tools for site-specific labeling of proteins in live cells, which is becoming increasingly important for SRM. These strategies involve genetically fusing a POI with a peptide tag or enzyme that can be selectively targeted by a fluorescent probe, allowing precise and efficient labeling.

One widely used approach utilizes self-labeling enzymes such as SNAP-tag, CLIP-tag, and HaloTag. The SNAP-tag, derived from human *O*^6^-alkylguanine-DNA alkyltransferase, reacts covalently with benzylguanine derivatives, while the HaloTag, a modified haloalkane dehalogenase, reacts with chloroalkane ligands (121). These enzymes enable the covalent attachment of synthetic fluorophores to the POI, offering flexibility in choosing dyes with optimal photophysical properties for SRM. They are particularly advantageous because they allow live-cell imaging without the need for cell fixation or permeabilization, preserving the native cellular environment (122). Despite their utility, self-labeling enzymes face similar limitations as FPs. Their relatively large size (∼20–30 kDa) may affect the function or localization of the POI, especially when fused to functionally critical domains (123). In addition, the need for exogenous substrates requires careful optimization of labeling conditions and washing steps to minimize background fluorescence from unreacted fluorescent probes (124). These considerations are particularly important in live-cell imaging, where high labeling specificity and minimal perturbation are essential. Recent works include exchangeable HaloTag ligands for SRI techniques (47) as well as particle tracking (125).

Common specialized epitope binding tags include the α-helix-forming ALFA-tag (126), the streptavidin-binding StrepTag (127), and the HA-tag derived from the influenza virus (128,129). Generally, peptide tag-based labeling strategies can also enable site-specific protein visualization to study protein localization and trafficking (6,130). These, often enzyme-mediated, labeling strategies, e.g., include the use of biotin ligase (BirA), which recognizes a specific 15-amino-acid acceptor peptide (AP-tag) and catalyzes the biotinylation of a lysine residue within this sequence (131). The biotinylated POI can then be detected using fluorescently labeled streptavidin. While this method provides site-specific labeling, the relatively large size of streptavidin (∼60 kDa) can introduce steric hindrance and increase linkage error, potentially affecting the accuracy of localization in SRM (6). Sortase A, a transpeptidase from *Staphylococcus aureus*, offers another enzyme-mediated labeling method. It recognizes the LPXTG motif at the C terminus of the POI and facilitates the attachment of labels containing oligoglycine residues (132). This technique allows for the attachment of various functional groups, including fluorophores, peptides, or even DNA strands for DNA-PAINT imaging (133). While Sortase A-mediated labeling offers versatility, it requires careful optimization to ensure specificity and efficiency of the labeling reaction. Phosphopantetheinyl transferase enzymes, such as Sfp or AcpS, can also be used to label POIs. These enzymes recognize small peptide tags (e.g., ybbR tag) and transfer CoA-linked substrates to the POI, allowing for site-specific labeling with various labels. This method provides flexibility in labeling but requires efficient enzyme expression and substrate delivery (134,135). Metal ion-assisted labeling exploits the affinity of certain peptide motifs for metal ions. For example, polyhistidine tags (His-tags) bind to nickel(II) ions coordinated to nitrilotriacetic acid (Ni-NTA). Fluorescent Ni-NTA derivatives can reversibly label His-tagged proteins (136). Although this method is straightforward and reversible, it may suffer from nonspecific binding and lower affinity compared with covalent labeling, which can limit its usefulness in high-resolution imaging (137). Lastly, direct conjugation of fluorescent probes to specific peptide motifs offers a minimalistic approach. As an example, the tetracysteine motif (CCXXCC) binds to biarsenical dyes such as FlAsH and ReAsH, which become fluorescent upon binding (138). While the small size of the tetracysteine tag is advantageous, these arsenic-based dyes can be cytotoxic, and nonspecific binding to endogenous cysteine-rich proteins may lead to background fluorescence and require stringent washing protocols (56).

In summary, protein tags and self-labeling enzymes enable precise and efficient labeling of POIs for SRM applications. The choice of tag and labeling strategy must balance factors such as tag size, potential impact on protein function, labeling efficiency, and specificity to ensure accurate and reliable imaging results. Continued advancements aim to minimize perturbations to the POI while maximizing labeling specificity, thereby enhancing the accuracy of super-resolution imaging.

### PAINT and DNA-PAINT

The reversible binding of peptides or oligonucleotides to biological structures using PAINT techniques has enriched SRM, enabling dynamic structural and molecular studies with nanometer precision (139). Both peptide PAINT and DNA-PAINT methods use docking strands integrated into the target proteins for reversible binding by fluorogenic imager strands or peptides, enabling continuous signal acquisition and minimizing photobleaching. While originally Nile Red-based low-affinity labels were used (139), recent innovations include the peptide PAINT technique, which uses optimized coiled coils for fast binding kinetics and minimal binding errors, as well as advances in DNA-PAINT, such as antibody-DNA conjugates and DNA barcoded labels, which improve multiplexing capabilities and imaging speed (76,140,141,142). Although high-affinity labels have been in the focus to enhance specificity and stoichiometric labeling, low-affinity labels have also shown to be particularly useful, especially showing reduced photobleaching (143), enabling multiplexing via label exchange (47) and extended imaging times in single-particle tracking (144).

### GCE

**Figure 2:**
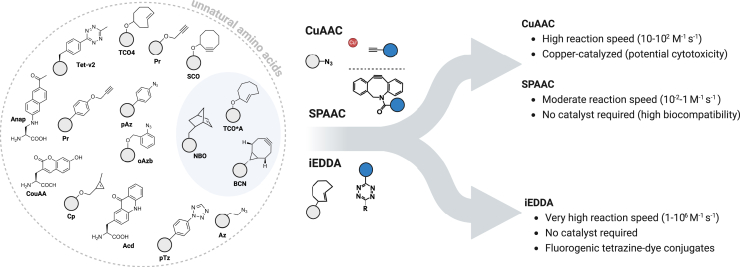
Overview of unnatural amino acids (uAAs) and chemical reactions for site-specific protein labeling. The most used clickable uAAs are highlighted in light blue. Arrows illustrate the chemical components of the most popular click reactions, along with their key features as reaction speed, catalyst requirements, and biocompatibility.

It is intuitively easy to understand that the smallest possible modification of a biomolecule would be the replacement or addition of a single building block of its kind. In the case of DNA or RNA, this would be a single basepair, in the case of the membrane, a single fatty acid, and finally in the case of proteins, a single amino acid. The combination of unnatural basepair transcription and posttranscriptional modification by click chemistry has enabled simple DNA and RNA labeling with a wide variety of functional groups at desired positions (145,146). Similarly, functionalized fatty acids have been widely used to label different lipids via click chemistry or directly conjugated to fluorophores (147,148,149). When it comes to proteins, GCE is a powerful approach that allows the site-specific incorporation of unnatural amino acids (uAAs) into proteins and peptides, providing unique, tailored chemical handles for precise labeling in SRM (63). This method enables the introduction of bioorthogonal reactive groups into POIs without significantly altering its structure or function. In living cells, GCE relies on engineered orthogonal pairs of transfer RNA (tRNA) and aminoacyl-tRNA synthetase (aaRS) that specifically incorporate an uAA in response to a specific codon, typically the amber stop codon (TAG) at a defined position during protein biosynthesis (150,151). The uAAs provide functional handles for site-specific conjugation of fluorophores via bioorthogonal chemistry, offering a highly controlled labeling mechanism ideal for conjugating fluorescent probes (152). These biorthogonals enable precise and selective labeling of specific targets in living cells without disrupting native cellular processes (Fig. 2). Notable among these are the strain-promoted azide-alkyne cycloaddition (SPAAC) and the inverse-electron-demand Diels-Alder cycloaddition (IEDDA). In SPAAC, the incorporation of *p*-azido-L-phenylalanine enables labeling with cyclooctyne-functionalized dyes without the requirement for copper catalysts, which can otherwise introduce cytotoxicity (9,10).

Meanwhile, IEDDA utilizes *trans*-cyclooctene-modified lysine to facilitate rapid and selective conjugation with tetrazine-functionalized fluorophores, making it particularly suited for live-cell imaging (153). Both reactions are highly specific and proceed with high reaction kinetics under physiological conditions, thus reducing potential interference with natural cellular activities. Moreover, the small size of the uAAs used in these reactions minimizes steric hindrance and linkage error, which is essential for achieving high spatial resolution in SRM (154).

Recent advancements in aaRS engineering, tRNA optimization and directed evolution have significantly improved the efficiency of uAA incorporation, achieving expression levels comparable with natural amino acids (155,156). In addition, the development of mutually orthogonal tRNA/aaRS pairs allows for the incorporation of multiple different uAAs simultaneously, facilitating multicolor imaging (157). Computational tools and databases have been developed to predict optimal sites for uAA incorporation, considering factors such as solvent accessibility and structural compatibility (158,159). Moreover, the use of fluorogenic probes in combination with GCE has enhanced the signal/noise ratio in imaging. Tetrazine-functionalized dyes can exhibit quenched fluorescence that is restored upon reaction with the uAA, enabling wash-free imaging and reducing background fluorescence (63,160). GCE has been successfully applied in various fields, including structural biology, neuroscience, and virology, where it has enabled direct observation of conformational dynamics of membrane proteins (161), visualization of synaptic proteins in their native neuronal environment (59,162), and mapping virus-host interactions without compromising infectivity (62).

In summary, GCE represents a versatile strategy for site-specific protein labeling. By combining GCE with bioorthogonal chemistry, researchers can achieve site-specific labeling with minimal linkage error, enhancing the capabilities of SRM to visualize cellular structures and dynamics at the molecular level. Continued development and optimization of GCE systems promise to expand the toolbox of site-specific and minimally invasive labeling, enabling deeper insights into complex biological processes.

### Organic dyes

Organic dyes are critical to SRM. These small-molecule-based fluorophores, which cover a spectrum ranging from the ultraviolet to the near-infrared region, are integral to all super-resolution techniques and are constantly evolving to meet the needs of specific applications (4). Their advantageous properties including minimal cytotoxicity, improved photostability, and customizable photomodulation, often outperform FPs and have made organic dyes indispensable for modern bioimaging (Table 2). The adaptability of organic dyes allows for custom modifications to improve their functionality, including tuning of spectral properties and enhancing photostability (163). As dyes are fundamentally linked to fluorescence labeling, the following section highlights the predominant classes of organic dyes and their importance and optimization for super-resolution applications.

**Table 2:** Dye classes, their properties, and applications in SRM

					
	BODIPYs	Coumarins	Cyanines	Oxazines	Xanthenes
**Property**					

Brightness	◒	●	●	●	●
Photostability	●	◒	◒	●	●
Cell permeability	◒	◒	◒	○	●
Environmental sensitivity	○	●	○	◒	◒

**e.g., used in**					

STED	Mitronova et al. (27); Zhu et al. (164)	Rankin et al. (165); Pajk et al. (166)	Sieber et al. (13)	Kessler et al. (167)	Arsić et al. (59); Willig et al. (15); Lukinavičius et al. (26)
SIM	Chen et al. (168); Ali et al. (169)		Gustafsson etal. (16)	Chen et al. (170)	Uttamapinant et al. (61); Nixon-Abell et al. (17); Huang et al. (32); Guo et al. (43); Han et al. (171)
*d*STORM	Adhikari et al. (36); Shim et al. (37)		Jungblut et al. (62); van de Linde et al. (19); Shim et al. (37); Helmerich et al. (172)	Beliu et al. (63); Wombacher et al. (35); Vogelsang et al. (173)	Takakura et al. (30); Beliu et al. (63); van de Linde et al. (19); Wang et al. (174)
DNA-PAINT			Youn et al. (133); Unterauer et al. (140); Steen et al. (175)	Kessler et al. (167); Ghosh et al. (176)	Steen et al. (175)
MINFLUX			Gwosch et al. (79); Remmel et al. (177)	Ostersehlt et al. (178)	Gerasimaitė ety al. (39); Schmidt et al. (179)
ExM	Wen et al. (180)		Tillberg et al. (46)		Chen et al. (23); Tillberg et al. (46)

This table summarizes five dye classes: BODIPYs, coumarins, cyanines, oxazines, and xanthenes—and their key properties are indicated by symbols (●, high; ◒, moderate; ○, low). The properties listed include brightness, photostability, cell permeability, and environmental sensitivity. References are given for SRM techniques, where they have been shown to be particularly useful.

Boron dipyrromethene (BODIPY) dyes are known for their high quantum yield and stability to environmental changes, making them prime candidates for protein and DNA labeling in SRM (181). Their structural adaptability allows for emission wavelength modifications, enhancing live-cell imaging applications. Despite solubility hurdles, their excellent cell permeability and neutral charge make them useful for all SRM techniques, including SMLM. Recent innovations have led to derivatives with improved photophysical properties, targeting specific cellular components and processes for detailed insights into cellular dynamics (182). Coumarin dyes, derived from natural coumarin, become fluorescent through specific substitutions, offering significant Stokes shifts and high quantum yields. Chemical optimization, allowing the creation of different photostable derivatives with reduced environmental sensitivity, have extended their applicability by improving resolution in live-cell imaging, although their application in SRM remains largely unexplored (183,184). Cyanine dyes, including Cy3, Cy5, and Alexa Fluor 647, are essential for biological studies due to their superior photophysical properties and photoswitching behavior (185,186). Their robust absorption and emission profiles, together with high fluorescence quantum yields, make them widely used in SRM. Innovations focused on reducing self-aggregation and developing water-soluble derivatives have minimized potential artifacts and improved the quality of super-resolution imaging (187,188,189). Oxazine dyes play an important role in both bioimaging and medicinal chemistry, with fluorophores such as ATTO 655 and NileRed known for their photostability and resistance to photobleaching. While their fluorescence quantum yield and cell permeability can pose challenges, novel derivatives aim to improve their brightness and live-cell compatibility, offering unique advantages for sensitive imaging applications (190). Xanthene dyes, which include fluoresceins, eosins, and rhodamines, are valued for their high fluorescence quantum yield and photostability. Suitable for a wide range of applications, innovations have resulted in derivatives with improved solubility, reduced cytotoxicity, and enhanced fluorescence, ensuring their broad applicability in SRM, including versatile imaging techniques and dynamic tracking of cellular processes (191). Silicon rhodamine (SiR) dyes are characterized by high fluorescence quantum yield and photostability, coupled with minimal cytotoxicity, making them particularly useful for live-cell imaging (192). Their extensive use in staining cytoskeletal components highlights their role in the dynamic observation of cellular architecture. Recent advances include novel derivatives with improved specificity and the development of photoactivatable and photoswitchable versions, enhancing their application in various SRM techniques (193,194,195).

## Conclusions and outlook

Advancements in fluorescent labeling strategies have profoundly impacted bioimaging, enabling exploration of biological systems at the molecular level. The continuous refinement of labeling techniques and the development of innovative fluorescent probes have not only diversified the tools available to researchers but have also pushed the boundaries of achievable resolution in light microscopy. The selection of an optimal labeling strategy is an important process, closely connected to the specific characteristics of the target biomolecule and the requirements of the chosen SRM technique. Antibodies and nanobodies have enabled precise epitope targeting without the need for cellular overexpression. While antibodies provide high specificity and diversity, their larger size can hinder access in densely packed molecular environments. Monobodies, with their smaller structure and robust stability, mitigate these spatial constraints but require careful consideration regarding target specificity and potential modification requirements. Smaller binders such as aptamers and affimers present cost-effective and highly stable alternatives, although challenges remain in affinity customization, target specificity, and commercial availability. Small organic probes offer target-specific binding with low linkage error, yet their application can be limited by inherent photophysical properties and modulation of biological functionality. Genetic labeling strategies, including FPs and self-labeling enzymes, have enabled direct visualization of proteins within their cellular context. While these approaches simplify imaging workflows, they may face limitations related to photophysical properties, potential interference with protein function, and, in the case of self-labeling enzymes, the necessity for substrate removal to minimize background signals. Advanced techniques like peptidePAINT and ultimately incorporation of reactive single amino acid anchors via GCE and provide exceptional precision and flexibility in labeling, facilitating detailed investigation of protein structures and dynamics with smallest linkage error. However, their successful application requires careful optimization and faces challenges such as complex genetic engineering, label availability, and multiplexing capabilities.

Looking to the future of fluorescent labeling in SRM, there is a clear trend toward the development of fluorescent markers specifically tailored for super-resolution techniques. The ability to visualize multiple targets simultaneously is becoming increasingly important to gain deeper insights into the complex interplay of cellular interactions and functions, making multicolor imaging capabilities critical. Recent developments in DNA barcoding technologies are proving to be powerful due to their multiplexing capabilities and improved throughput, achieved by decoupling the DNA barcoding of target proteins from the imaging process (140). However, these technologies currently mainly rely on structure-affinity binders, which come with their abovementioned limitations. Combining this strategy with novel labeling approaches, e.g., GCE and bioorthogonal click chemistry, will be very powerful for dissecting the molecular interplay of biomolecules, not only in the field of bioimaging but also related fields such as spatial omics. One approach might be the simultaneous incorporation of distinct uAAs in proteins via multiple, mutually orthogonal tRNA/aaRS pairs. Each engineered tRNA/aaRS pair recognizes only a specific uAA, ensuring bioorthogonality across the endogenous and engineered tRNA/aaRS pairs (196). Although it is still challenging to introduce more than four uAAs via classical codon reassignment into living cells, latest developments in tRNA engineering (using, e.g., quadruple or quintuple orthogonal tRNAs) could pave the way for simultaneous introduction of multiple uAAs into POI (197,198,199). In the end, only the interdependence between introduction of uniquely addressable uAAs with mutually orthogonal chemical reactivities will ultimately limit the multiplexing capacity of this labeling strategy.

Further innovation is required to keep a balance between highly specific labeling approaches and minimal physiological perturbation. The here discussed limitations such as large linkage error or genetic modification of the POI contain the risk to alter protein functions, hinder binding partners or perturb the localization precision. While there are potential solutions in ultrasmall labels (e.g., GCE) or optimized orthogonal chemistries for biological questions (10), this area still requires additional examination. The continued evolution of SRM techniques reaching molecular precision underscores the critical dependence of innovations in labeling strategies. Ultimately, it will be the precision and specificity of labeling strategies that enhance fluorescence microscopy and allow the visualization of molecular details.

## Data availability

No data were used for the research described in the article.

## Acknowledgments

We apologize to the authors of any significant contributions not cited here due to space limitations and acknowledge the extensive work that has shaped the field of fluorescence labeling and bioimaging. This project is supported by the Federal Ministry for Economic Affairs and Climate Action (BMWK) on the basis of a decision by the German Bundestag (Grant Agreement No. KK5665801HV4 to G.B.) All figure panels were created with biorender.com.

## Declaration of interests

The authors declare that they have no known competing financial interests or personal relationships that could have appeared to influence the work reported in this paper.
